# The Patient Monitoring Roundtable as Catalyst for Health Care Innovation: Case Study

**DOI:** 10.2196/82786

**Published:** 2026-04-01

**Authors:** Elena Hinz, Jasper Wagnitz, Merve Sarica, Anne Rike Flint, Amin Chaoui, Mona Prendke, Louis Agha-Mir-Salim, Felix Balzer, Akira-Sebastian Poncette

**Affiliations:** 1 Initiative for Innovation and Collaboration in Healthcare (INCH) e.V, Berlin, Germany; 2 Institute of Medical Informatics, Charité - Universitätsmedizin Berlin, Corporate member of Freie Universität Berlin and Humboldt-Universität zu Berlin, Charitéplatz 1, Berlin, 10117, Germany, 49 30450631; 3 Einstein Center Digital Future, Berlin, Germany; 4 Department of Anesthesiology and Intensive Care Medicine, Charité - Universitätsmedizin Berlin, Corporate member of Freie Universität Berlin, Humboldt-Universität zu Berlin, Berlin, Germany

**Keywords:** digital health, transdisciplinary research, patient-centered care, health care innovation, medtech, ehealth

## Abstract

**Background:**

In the field of patient monitoring, there often remains a gap between clinical needs and the monitoring technologies available from industry. To conquer this, the Patient Monitoring Roundtable (PMRT) live event series offers a sustainable and structured platform for innovation through focused small-group discussions, prioritizing deep engagement among stakeholders. By establishing a dynamic, low-barrier forum, the PMRT aims to serve as a thought leadership platform in patient monitoring and digital health, driving continuous improvement and shaping the future of health care technology.

**Objective:**

This paper pursues 2 main objectives: first, to describe the concept, implementation, and practical insights of the PMRT as a novel format for transdisciplinary collaboration in digital health; second, to evaluate its perceived impact and reception among participants.

**Methods:**

The concept and implementation of the format were described using internal planning documents, event materials, and communication records. To evaluate participant reception and perceived impact, a cross-sectional online survey was conducted between October 2024 and January 2025. The questionnaire was distributed at PMRT events and via digital channels, including the PMRT newsletter and LinkedIn.

**Results:**

The PMRT was conducted 29 times between January 2022 and June 2025. It is usually structured in a keynote, followed by interactive small group workshops and a consecutive group discussion, and concluded by a networking session. Examples of topics include alarm management, tele-surveillance and care, user testing of monitoring devices, implementation science, data protection and cybersecurity, artificial intelligence in medicine, and interoperability. Following each event, a structured set of postevent activities ensures continued engagement and knowledge dissemination. A total of 47 responses were included in the survey analysis. The 2 most represented professional backgrounds were industry representatives and physicians. Other participants came from nursing, research, or other fields. Most respondents reported having experience in patient monitoring and digital health, with no notable differences across professional backgrounds or gender. More than half had attended several PMRT events. Formats such as small group discussions and workshops were viewed as highly effective in fostering dialogue. Most participants felt they were part of a professional community, and some reported having established new collaborations. Informal exchanges and interactive workshops were seen as the most valuable aspects of the event.

**Conclusions:**

The PMRT presents a novel, structured, low-threshold platform for clinical-centered transdisciplinary dialogue, professional networking, and knowledge exchange in the field of digital health. Participant reception confirms high acceptance and perceived impact, with many reporting strengthened community ties and new collaborations and valuing the interactive workshop format. These findings demonstrate that the PMRT can serve both as an innovative collaboration model and as a platform with tangible benefits for its attendees.

## Introduction

Patient monitoring systems are essential for the continuous assessment of patients’ physiological status in high-acuity environments such as intensive care units, operating theaters, and emergency departments and are increasingly used on general wards to enable early detection of patient deterioration [1,2]. Over the past decades, advances in sensor technology, data integration, and analytic capabilities have expanded the scope and complexity of monitoring. Nevertheless, there is often a gap between clinical needs and the technologies available from industry [3-5]. Common challenges include alarm fatigue from excessive and often nonactionable alerts, fragmented device ecosystems lacking interoperability, and usability issues that hinder integration into clinical workflows [6-8]. Addressing user needs is critical for the successful adoption of medicotechnical innovations, as misaligned solutions often remain unused or create barriers in daily workflows [9-14]. Early and continuous user involvement is therefore essential to achieving real-world impact.

Hackathons have the potential to address this gap by fostering early, collaborative problem-solving among stakeholders while accelerating innovation and fostering transdisciplinary collaboration. Originating in the tech industry, these events unite clinicians, engineers, designers, industry representatives, and patients to co-create practical solutions [15]. However, prior research shows that hackathons demand substantial resources and frequently struggle to sustain momentum or translate ideas into long-term impact [16-19].

Building on the collaborative, user-centered, and solution-oriented spirit while addressing their limitations in sustainability and follow-up, we developed the Patient Monitoring Roundtable (PMRT). In the context of the PMRT, “patient monitoring” encompasses both established clinical monitoring systems, including traditional bedside monitoring of vital signs, and emerging digital health technologies and data-driven tools that collect, integrate, and interpret physiological and clinical information to inform care practices and workflows. The PMRT in the form of a live event series offers a sustainable and regular platform for innovation through focused, small-group discussions, prioritizing deep engagement among stakeholders. By establishing a dynamic, low-barrier forum, PMRTs aim to create a community of like-minded experts, driving continuous improvement and shaping the future of patient monitoring and digital health.

Against this background, this paper pursues 2 objectives. First, it describes the concept, implementation, practical insights, and outcomes of the PMRT events as a novel format for interdisciplinary collaboration in digital health. Second, it presents findings from a participant survey that evaluated the perceived impact and reception of the PMRT, acknowledging the challenges of measuring open exchange formats. Through this initiative, we aim to advance health care innovation in a manner that is both impactful and sustainable.

## Methods

### Origin

The PMRT was initiated under the framework of Hacking Health, a global initiative aimed at fostering innovation in health care by bringing together diverse stakeholders, such as clinicians, industry representatives, and researchers, to collaborate on solving pressing medical challenges through hackathons, workshops, and community events. The Berlin branch of Hacking Health was founded in 2014, supported by Hacking Health Canada [16]. As part of its efforts to promote user-driven innovation, the Berlin branch organized its first health hackathon in February 2017. Over the course of 2 days, participants worked in transdisciplinary teams to develop digital health solutions, while researchers conducted a field study to capture insights on collaboration dynamics and innovation processes. Building on this experience, two additional health care hackathons were conducted in December 2017 and November 2018.

### Development of the PMRT

In a series of interdisciplinary meetings, a group of researchers and students consisting of ASP, MS, JW, and LA-M-S created the concept of the first PMRT, drawing on lessons from prior hackathon experiences. In January 2022, the first event was conducted, and the series was established to promote an ongoing low-threshold exchange of pain points, ideas, and feedback with a focus on the topics of patient monitoring and digital health. Since then, the PMRT has evolved into a structured event series supported by a network of sponsors such as Masimo [20], Dräger [21], and Philips [22] and partners such as HealthCapital [23], the Berlin Institute of Health (BIH) [24], and the Einstein Center for Digital Future (ECDF) [25].

The target audience encompasses individuals from a variety of professional backgrounds, mainly including clinicians such as nurses and physicians; hospital IT staff and industry representatives; but also patient representatives, scientists, and students. A special focus lies on the exchange between the clinical practice and patient monitoring device manufacturers.

As of June 2025, a total of 29 roundtable events have been conducted in Berlin, Germany, and partly via an online approach. The organizing team eventually expanded to 5 members, all of whom were part of the nonprofit organization Initiative for Innovation and Collaboration in Healthcare (INCH) e.V., as a successor of Hacking Health Berlin.

While the PMRT is supported by industry sponsors, the format is designed as a co-creative platform involving multiple stakeholder groups. Industry partners may propose topics and contribute to the co-design of selected events, with final selection made by mutual agreement. As part of the governance framework, sponsoring partners are explicitly instructed to limit promotional content and to ensure that their contributions are framed in a way that provides clear value for clinical participants. Final decisions regarding topic selection, agenda structure, and workshop formats rest with the organizing team. Sessions are primarily moderated by members of the organizing team or independent experts, for example, with affiliations to universities or professional societies. In rare cases, industry experts have contributed to moderation within the same governance framework. In addition, some PMRT events have included clearly delineated, product-focused sessions. These sessions were embedded within a broader clinical and methodological discussion and did not constitute the dominant format of the event.

### Data Collection and Analysis

The structure, development, and implementation of the PMRT format were reconstructed and documented using multiple internal sources, including planning documents (eg, agendas, timelines, and stakeholder lists), event materials (eg, programs, presentations, and workshop templates), and communication records (eg, meeting notes and email correspondence).

To evaluate the impact and reception of the PMRT, a cross-sectional online survey was conducted using an online questionnaire (Multimedia Appendix 1). The survey instrument was designed as a pragmatic evaluation tool to assess participant experiences and the perceived impact of the PMRT format. Item generation was informed by the objectives of the roundtable series and recurring themes from prior events. The questionnaire was reviewed internally by the organizing team to ensure clarity and relevance prior to use. Minor refinements were made iteratively based on this early feedback. No formal psychometric validation procedures (eg, test-retest reliability or construct validation) were conducted prior to deployment. Potential participants were defined as people who have attended at least one roundtable event and are not involved in its organization or conduction. The questionnaire was administered during 3 roundtable events; distributed via the PMRT newsletter, which had 289 subscribers as of July 7, 2025; and a call for participation on LinkedIn (Microsoft Corp). It was accessible for submission between October 15, 2024, and January 31, 2025.

Data were collected regarding the participants’ professional background, experience and satisfaction, results and impact, criticism, and suggestions in regard to the PMRT. Answer formats included nominal scales, ranked-order questions, 5-point Likert scales (5=“very applicable,” 4=“rather applicable,” 3=neutral, 2=“rather not applicable,” 1=“not applicable at all”) and free-text responses. In cases of incomplete questionnaires, only the answered items were considered for analysis. Results were analyzed using Microsoft Forms and IBM SPSS Statistics (version 29.0.2.0). Absolute and relative frequencies were calculated, and the median was reported for ordinal-scaled questions. Additionally, correlations between responses and professional groups were examined for selected questions using the Fisher exact test, given the small sample size and the categorical nature of the data with low expected frequencies. All reported *P* values refer to this test. The survey instrument was developed, administered, and analyzed independently by the organizing team. Sponsors had no access to raw survey data and no influence on data analysis or interpretation.

### Ethical Considerations

The study received approval from the institutional ethics committee of Charité–Universitätsmedizin Berlin (EA1_055_25). Participants received information about the study prior to participation and provided informed consent before taking part. Participation was voluntary, and no financial compensation was provided. All data were collected, stored, and processed in accordance with institutional guidelines and the General Data Protection Regulation (GDPR). Only pseudonymized data were used for analysis.

## Results

The following results section comprises 2 parts, first, a description of the concept, implementation, and practical insights of the PMRT live event; and second, findings from an online survey evaluating its perceived impact and reception among participants.

### Patient Monitoring Roundtable Live-Event

#### Roundtable Preparation

The successful execution of each roundtable requires a thoroughly coordinated preparation phase, encompassing the scheduling of dates and venues, the selection of relevant topics, the application for continued education points, and the implementation of a targeted promotional strategy. These processes are described in detail in the following section.

##### Scheduling

The roundtables are scheduled 8 times annually, evenly distributed across the calendar year. A key lesson learned was that no events should be scheduled in July, August, or December, as the summer and Christmas holiday periods significantly reduce participant availability. At participants’ request, event dates are planned at the end of the preceding year and are then confirmed 3 months in advance to accommodate the scheduling constraints of clinicians and other participants. Each roundtable is held on a weekday evening and lasts 2.5 hours (6:00 PM-8:30 PM).

##### Location

To facilitate accessibility and encourage attendance, events are hosted at centrally located venues in Berlin close to hospital facilities, with locations varying across events depending on availability and suitability for the planned format. Requirements for venues include a projector and a larger room for keynotes and group discussion, smaller rooms for breakout sessions, and Wi-Fi for hybrid sessions.

##### Topic Selection

Over the course of the PMRT events, it became evident that topic selection is most effective when combining several sources, including suggestions from participants grounded in their day-to-day experience with patient monitoring, emerging trends in scientific research, input from industry partners, and the expertise of potential keynote speakers. These inputs are jointly reviewed and prioritized by the organizing team to ensure that each event addresses challenges and developments of direct relevance to the audience.

##### Continuing Education Points for Nurses and Physicians

In Germany, proof of continued training is mandatory for medical specialists, and a certain number of so-called Continuing Medical Education points [26] must be collected. Since January 2025, we have applied and received Continuing Medical Education certification for each roundtable. A similar system exists for nursing staff, who are required to collect so-called nursing points to continue their facultative registration [27]. Since April 2025, we have also applied regularly for these certifications.

##### Promotion and Registration

A comprehensive promotional strategy was implemented, using the PMRT’s official website [28] and newsletter, partner organizations’ online platforms, and social media channels such as LinkedIn and Instagram (Meta Platforms). Event registrations are available free of charge and are managed through Eventbrite (Eventbrite Inc) [29], a global platform for ticketing and event organization. The number of registrations, that is, tickets, available mainly depends on the capacity of the event location and the intended agenda. A lesson learned in 2025 was to introduce separate registration contingents for clinicians and other professionals to ensure balanced representation of both groups. No systematic tracking of outreach channel effectiveness for event registrations (eg, referral links or click-through analytics) was conducted; based on organizer observations, most registrations appeared to follow newsletter announcements, and intranet postings often prompted registrations among clinical participants.

### Roundtable Event

#### Topics and Participants’ Interests

Depending on the topic, each PMRT event is co-designed and co-conducted with renowned experts in the respective field to ensure relevance, depth, and practical impact. Examples of topics include alarm management, tele-surveillance and -care, user testing of monitoring devices and medical smartwatches, user experience design in alarm management, monitoring in weightlessness, implementation science, data protection, and cybersecurity, artificial intelligence in medicine, and interoperability. Experience has shown that participants are especially interested in topics that are prominently discussed in general societal discussion, such as artificial intelligence and cybersecurity or are directly linked to their daily professional life, such as alarm management and monitoring devices. A complete list of topics, formats, and estimated number of participants can be found in Multimedia Appendix 2.

#### Event Structure

**Figure 1.**
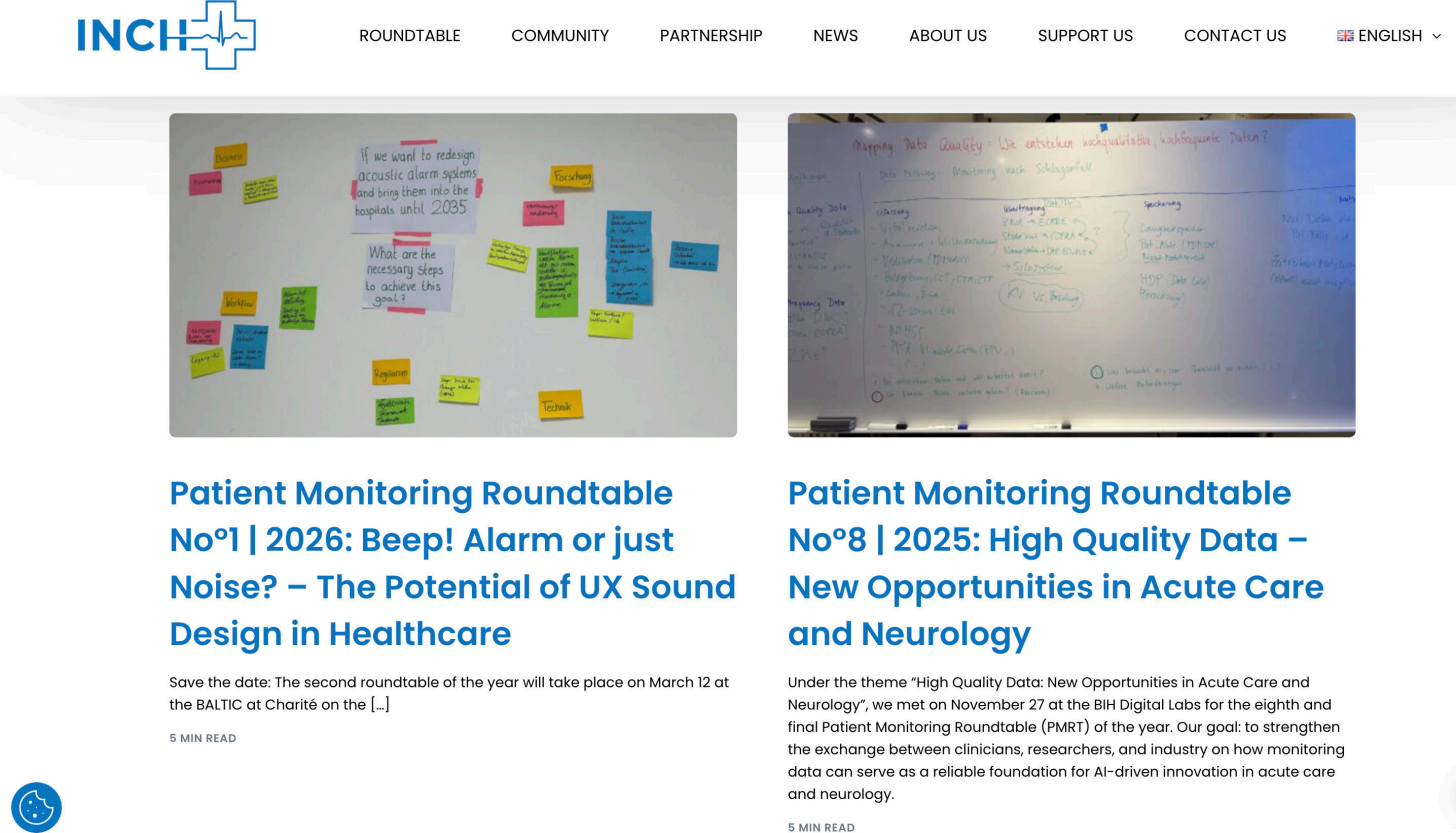
An example of 2 blog posts summarizing recent Patient Monitoring Roundtable (PMRT) events on the Initiative for Innovation and Collaboration in Healthcare (INCH) website [31].

From the outset, each roundtable has used a variety of formats, including keynote presentations, panel discussions, World Cafes [30], small group discussions, and hands-on workshops. Especially the combination of starting the event with an introductory keynote followed by interactive workshops in small groups has been established. Sessions conclude with a group discussion followed by a networking session, providing participants with the opportunity to establish and strengthen professional connections. The contribution of expertise and personal experience is strongly encouraged to promote meaningful dialogue and cross-sector collaboration in problem-solving. Keynotes are recorded using smartphones and clip-on microphones. Since 2025, a hybrid event solution with Microsoft Teams has been introduced, where possible, to accommodate virtual attendees and enhance accessibility. Online participants have the opportunity to submit questions via the chat function. Following the keynote, an additional online workshop is held in parallel to the in-person breakout groups. During the final group discussion, online participants can again contribute via the chat. A typical agenda is displayed in Figure 1 [31].

#### Moderation

Each event starts with opening words by the PMRT team, introducing the topic, today’s agenda, and potential keynote speakers. Depending on the content and the experts involved, the workshops are led either by the PMRT team or by external experts. The discussion in the large group format and concluding remarks are again facilitated by the PMRT team. While most events are held in German, the roundtable is occasionally conducted in English, depending on the language preferences of the experts and the language profile of the audience.

### Postevent Activities

#### Overview

**Figure 2.**
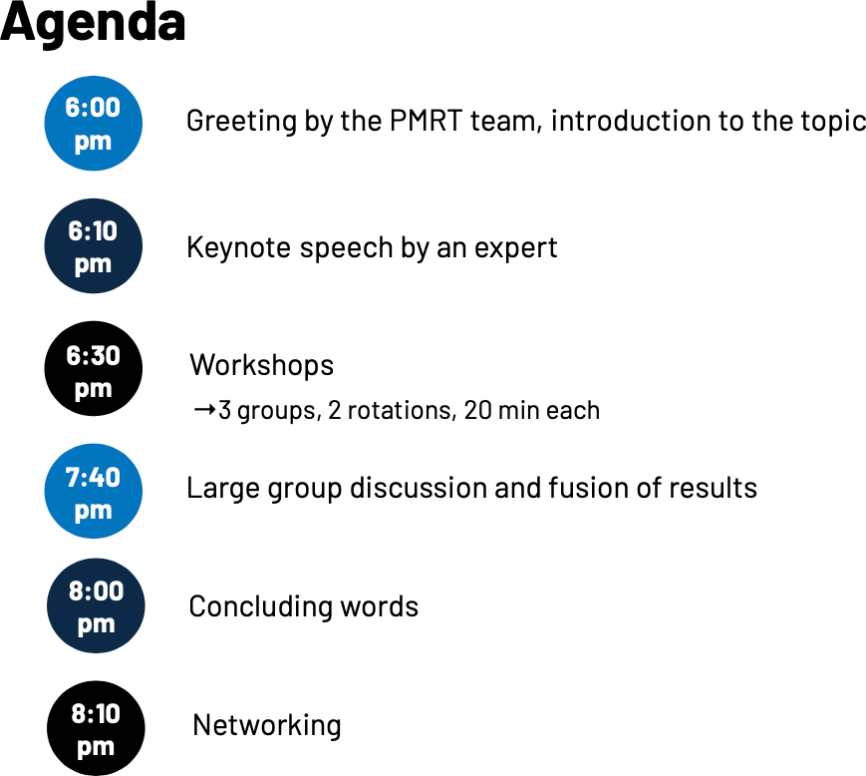
A typical agenda of the Patient Monitoring Roundtable (PMRT) starts with a short introduction and keynote at 6 PM, followed by an extensive workshop session and subsequent discussion lasting about 2 hours, concluded by a networking session. PMRT: Patient Monitoring Roundtable.

Following each event, a structured set of postevent activities ensures continued engagement and knowledge dissemination. A newsletter summarizing the event, including a brief description, key takeaways, and a link to the keynote video, and an announcement for the next roundtable are distributed across multiple channels. This includes email newsletters and social media platforms such as LinkedIn and Instagram, as well as institutional communication channels such as the Charité intranet and the websites of the Institute of Medical Informatics at Charité-Universitätsmedizin Berlin [32] as well as INCH e.V [28] (Figure 2).

#### Participant Engagement and Event Attendance Trends

Participants of the first 6 events in 2022 were invited exclusively via email, primarily targeting industry representatives, clinicians (including nurses and physicians), and researchers with direct or indirect connections to the organizing team. Over time, as the event series became more established, personal invitations via email decreased in favor of registrations through Eventbrite. Registration data are available starting from September 2022. For the 2 recorded events that year, an average of 20 registrations was recorded. In 2023, this average increased to 27 in the first half and 29 in the second half. The upward trend continued in 2024, with the first 4 roundtables averaging 38 registered participants and the following 4 averaging 61. In 2025, the first 5 roundtables recorded an average of 54 in-person registrations and 17 online registrations, reflecting the continued growth and hybrid nature of the format. Total attendance was not consistently recorded across all events.

### Survey Results

#### Study Population Characteristics

As the survey was distributed via QR code during PMRT events and shared on LinkedIn, no exact response rate can be determined. However, considering that each event typically attracts around 40 participants on site, the number of responses represents a substantial share of the actively engaged audience.

Regarding demographics, the majority of participants identified as male, with most respondents falling into the 30‐39 years and 50‐59 years age groups. Industry representatives and physicians were the largest professional groups, making up nearly half and a quarter of the sample, respectively. Smaller proportions included nurses, researchers, and specialists from fields such as information technology, user experience research, and innovation management (see Table 1).

**Figure 3.**
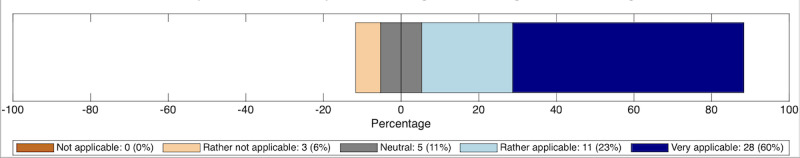
Distribution of participants’ responses to the statement “I have experience in the field of patient monitoring and/or other digital health technologies.” Percentages indicate the proportions of answers given per Likert item (eg, “rather applicable”); n=47. PMRT: Patient Monitoring Roundtable.

The majority of participants reported having experience in patient monitoring and/or other digital health technologies, with the median response to the statement, “I have experience in the field of patient monitoring and/or other digital health technologies,” being “very applicable” on a 5-point Likert scale (Figure 3). Further analysis by professional group (clinical vs industry vs other) showed no statistically significant differences (Fisher exact test, *P*=.11). Similarly, no significant differences were found when analyzing responses by gender (Fisher exact test, *P*=.15).

**Table 1.** Study population characteristics.

Characteristic	Values, n (%)	
Gender		
Man	31 (66)	
Woman	15 (32)	
Not disclosed	1 (2)	
Age group (years)		
18‐29	7 (15)	
30‐39	19 (40)	
40‐49	9 (19)	
50‐59	11 (23)	
>60	1 (2)	
Professional background		
Industry representatives	21 (45)	
Physicians	11 (23)	
Health and nursing professionals	4 (9)	
Researchers	5 (11)	
Other^a^	6 (13)	

a Includes hospital information technology specialists, user experience researchers for medical hardware and software, cofounders with a focus on user experience research, innovation managers, designers, and physician-scientists.

#### Engagement With PMRT Events

More than half of the respondents (25/47, 53%) reported having attended four or more PMRT events, 9 out of 47 participants (19%) have attended 2‐3 events, while 13 out of 47 (27%) have participated in the roundtable only once. There is no significant correlation between the professional background and the number of attended events (*P*=.19).

**Figure 4.**
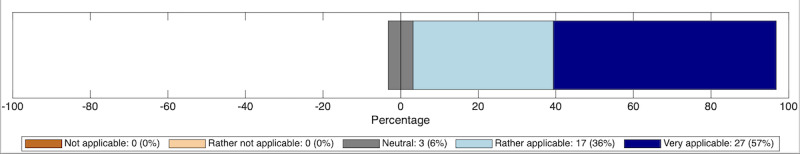
Distribution of participants’ responses to the statement “I have already recommended, or would recommend, the PMRT to a colleague” (n=47). Percentages indicate the proportions of answers given per Likert item (eg, “rather applicable”). Percentages may not sum to 100 due to rounding. PMRT: Patient Monitoring Roundtable.

Around 44 (94%) out of 47 respondents indicated that it would be very likely or rather likely that they would recommend the PMRT to colleagues. A total of 3 out of 47 (6%) remained neutral (Figure 4). Analyzing the responses by professional group did not yield statistically significant differences (*P*=.08).

#### Relevance and Impact of PMRT Events

**Figure 5.**
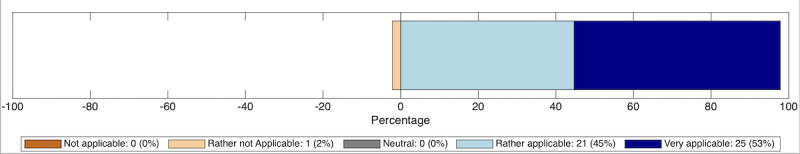
Distribution of participants’ responses to the statement “The content of the PMRT events I have attended is relevant to my professional interests” (n=47). Percentages indicate the proportions of answers given per Likert item (eg, “rather applicable”). PMRT: Patient Monitoring Roundtable.

Regarding the relevance of the event content, the majority of participants found the PMRT content very or rather applicable to their professional interests (46/47, 98%). One respondent stated that the content was not relevant. The median response was “very applicable” (Figure 5). An analysis by a professional group showed no statistically significant differences (*P*=.95), indicating no systematic difference in responses across different professional backgrounds.

**Figure 6.**
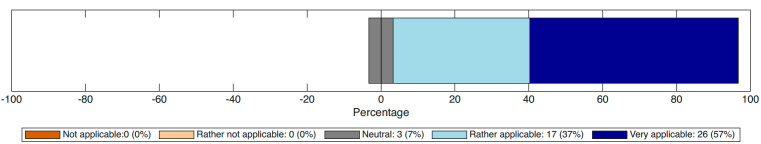
Distribution of participants’ responses to the statement “The formats of the PMRT events (eg, small group discussions and workshops) effectively promote dialogue among participants” (n=46). Percentages indicate the proportions of answers given per Likert item (eg, “rather applicable”). Percentages may not sum to 100 due to rounding. PMRT: Patient Monitoring Roundtable.

Similarly, the event formats, such as small group discussions and workshops, are widely regarded as effective in promoting dialogue among participants. Around 43 out of 47 attendees (93%) rated them as very effective or rather effective, 3 remained neutral, and one did not answer (Figure 6).

**Figure 7.**
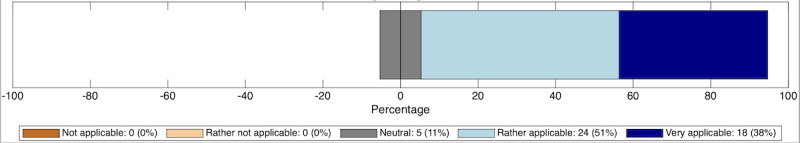
Distribution of participants’ responses to the statement “Participating in the PMRT gives me the feeling of being part of a community of like-minded professionals in the field of patient monitoring and digital health” (n=47). Percentages indicate the proportions of answers given per Likert item (eg, “rather applicable”). PMRT: Patient Monitoring Roundtable.

Many respondents reported feeling connected to like-minded colleagues, with most describing this as very or rather applicable (42/47, 89%). A smaller number of participants expressed neutrality on this point (5/47, 11%). The median response was “rather applicable” (Figure 7).

When asked whether attending one or more PMRT events led to new collaborations, 13 out of 47 participants (28%) responded with “yes,” while an equal number (28%) stated “no.” Additionally, 21 out of 47 respondents (45%) indicated that they had not yet entered a collaboration but intended to do so.

Nine participants gave further details on the kind of collaboration. One innovation manager established connections with PMRT partners and various companies. Three industry representatives and one researcher developed new business contacts, while another industry representative initiated a collaboration within the PMRT network. Further collaborations included one industry representative who connected with a startup and another who started a project with nursing staff. Additionally, one physician engaged in a research collaboration.

**Figure 8.**
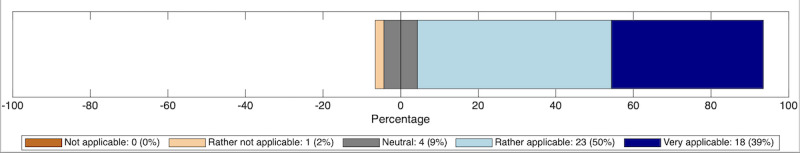
Distribution of participants’ responses to the statement “Attending one or more PMRT events has provided me with new insights that I would not have gained otherwise” (n=46). Percentages indicate the proportions of answers given per Likert item (eg, “rather applicable”). PMRT: Patient Monitoring Roundtable.

**Figure 9.**
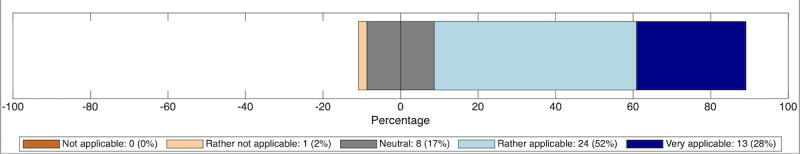
Distribution of participants’ responses to the statement “I will apply new insights or knowledge gained through the PMRT in my professional work” (n=46). Percentages indicate the proportions of answers given per Likert item (eg, “rather applicable”). Percentages may not sum to 100 due to rounding. PMRT: Patient Monitoring Roundtable.

Regarding whether attending the PMRT events provided new insights that would not have been gained otherwise (Figure 8), 43 respondents found this to be “very applicable” or “rather applicable.” Three participants (6%) remained neutral. One response (2%) was left unanswered. The median response was “rather applicable.” The statement “I will apply new insights or knowledge gained through the PMRT in my professional work” was assessed by 46 participants. Out of 47 participants, one respondent (2%) did not answer. One respondent (2%) indicated that the statement was rather inapplicable. Eight participants (17%) were neutral, whereas 24 respondents (51%) considered it rather applicable, and 13 participants (28%) rated it as highly applicable. An analysis by a professional group revealed no significant differences (*P*=.37). The median response was “rather applicable” (Figure 9).

#### Feedback and Suggestions

Participants were asked to rank the most relevant aspects of the PMRT in order of importance. Of the 47 participants, 43 answered the question, while 4 did not provide information. The informal exchange with clinicians and manufacturers was rated as the most relevant aspect, with 25 (57%) of respondents ranking it as their top priority. This was followed by interactive workshops, which were most relevant for 9 (20%) participants and ranked second by 14 (32%). Short keynotes received more mixed responses, with only 8 (18%) ranking them as most relevant, while 12 (27%) placed them in the lowest priority category. The presence of renowned experts was seen as highly relevant by only 2 (5%) respondents, but 14 (32%) ranked it as the least important aspect. Small group discussions were rated as the top priority by 6 (14%) of participants, while the majority (16/47, 36%) ranked them in the middle. A further breakdown of the results by professional group did not reveal significant differences in preferences. The question regarding barriers to participation in PMRT events revealed several challenges faced by respondents. The most frequently mentioned obstacle was scheduling conflicts, cited by 30 participants, accounting for 60% of responses. Financial reasons related to travel cost were also a notable barrier, affecting 10 participants (20%).

## Discussion

### Principal Findings

The PMRT presents a novel, practice-oriented format to foster transdisciplinary exchange and user-centered innovation in the field of patient monitoring and digital health, facilitated through the methodological approach of combining structured event planning with iterative community engagement. The steady increase in event participation, from 20 registrations in 2022 to over 60 per event in 2025, underscores the growing relevance and perceived value of the series. One of the key strengths of the PMRT format lies in its low-threshold accessibility, which has been further enhanced by the integration of hybrid elements since 2025. In methodological terms, the iterative and adaptive character of the PMRT allows for real-time responsiveness to participant feedback and emerging needs. Yet, this flexibility also limits the generalizability of insights beyond the specific context of Berlin and the partner institutions involved.

Based on three years of iterative implementation, several practical insights have emerged that may guide others seeking to establish similar transdisciplinary, practice-oriented formats. These insights reflect the real-world dynamics of engaging diverse stakeholder groups in patient monitoring and digital health.

### Key Lessons Learned

The following principles should be considered when designing and implementing the program:

- Ensure relevance: choose topics that closely reflect participants’ professional realities to maximize practical value and engagement.
- Maintain continuity: establish a regular event rhythm to build trust, foster relationships, and sustain participation over time.
- Foster true transdisciplinary: involve diverse stakeholder groups so that each gains tangible benefits from the exchange.
- Use engaging formats: combine concise, high-value keynotes with interactive small-group workshops to stimulate discussion and collaboration.
- Implement clear roles: move from ad-hoc responsibilities to clearly defined, specialized roles to improve efficiency and ensure consistent quality.

### Understanding Participants’ Perspectives

Beyond the quantitative overview, the survey results provide deeper insights into the composition of the PMRT community, their engagement patterns, and perceived outcomes. Nearly half of participants were industry representatives, a proportion that is consistent with observations from the PMRT organization, where this group appeared more inclined to participate than clinical professionals. This likely reflects differences in workload, institutional constraints, and the prioritization of networking opportunities, as well as the more immediate benefits industry stakeholders can derive from qualified feedback. In contrast, the incentives for clinicians may be less tangible. While the roundtable already attracts a diverse audience, future efforts will focus on achieving greater gender balance and increasing participation from health care professionals, and particularly nurses, to better reflect the diversity of perspectives in real-world patient monitoring.

More than half of the respondents had attended four or more events, indicating strong and continued interest. The high likelihood of recommending the PMRT to colleagues further supports the perceived value of the platform. Interestingly, no significant differences in engagement were observed between professional groups.

The event formats were widely regarded as effective in fostering dialogue and participation in PMRT events contributed to a strong sense of professional community, which is a particularly important outcome, as transdisciplinary collaboration is often cited as a critical factor in advancing patient monitoring technologies.

Beyond fostering discussions, the PMRT has effects on professional networking and collaboration. More than a quarter of respondents reported having initiated new collaborations as a direct result of attending PMRT events, including industry partnerships, business contacts, startup engagement, and research projects, while almost half of the participants indicated an intention to collaborate in the future. On one hand, this highlights the PMRT’s role not only as a platform for knowledge exchange but also as a catalyst for transdisciplinary cooperation. On the other hand, these collaborations are often one-off and not the kind that supports ongoing innovation.

While the overall reception of the PMRT was highly positive, certain barriers to participation were identified. Scheduling conflicts emerged as the most frequently cited challenge, affecting 30 out of 47 (60%) respondents. This issue will hopefully partly resolve with the introduction of a hybrid solution. Financial constraints were another notable barrier, impacting one-fifth of participants. As of today, the PMRT is free of charge. However, participants without residence in the area need to cover travel expenses. Other obstacles, such as technical difficulties and perceived lack of relevant content, were reported less frequently but still warrant consideration.

The PMRT is intentionally designed as a cross-sector forum that includes medical device manufacturers and is supported by industry sponsors. While this enables direct dialogue and practical feedback loops between clinicians and industry partners, it also introduces a structural risk of positive bias in participant-reported outcomes, particularly given the comparatively high proportion of industry respondents in our sample. Consequently, the results should be interpreted as perceptions of value and usefulness among engaged participants rather than as independent evidence of effectiveness.

Future evaluations should aim for independent replication, proactive recruitment of clinical stakeholders (including nursing professionals), and the inclusion of more objective indicators (eg, documented follow-up projects, implementation activities, or longitudinal tracking of collaborations).

### Positioning the PMRT in the Context of Innovation Frameworks

To contextualize the PMRT within established innovation models, we draw on the quintuple helix framework [33], which has been widely adopted to analyze innovation processes in knowledge societies, emphasizing the interplay of ecological, social, and cultural dimensions alongside traditional institutional actors [34,35]. Unlike earlier models such as the triple and quadruple helix, which focus primarily on academia, industry, government, and civil society [36,37], the quintuple helix explicitly recognizes the natural environment as a key driver of innovation. The PMRT exemplifies a targeted application of the quintuple helix framework in health technology innovation, emphasizing multiactor collaboration while revealing persistent gaps.

In line with the literature, the PMRT exhibits strong engagement with the academic helix, as it actively addresses all major components, namely teaching and education, research, and initiatives such as innovation, democracy, and civic education [38]. The industry helix benefits from active participation by manufacturers, enabling practical insights into scalable implementation, an approach highlighted in recent studies as critical for bridging the gap between research and market adoption [39,40].

However, the political-regulatory helix remains underrepresented, reflecting broader challenges in embedding governance actors into innovation ecosystems. Although clinical staff across hierarchies and specialties actively represent user interests (aligning with the civil society helix), direct patient involvement remains limited. Ecological impacts are indirect, primarily through optimized technology deployment reducing implementation waste.

Overall, the PMRT aligns with the Quintuple Helix model’s emphasis on multiactor collaboration and knowledge integration, yet also exemplifies the persistent underrepresentation of regulatory, civil society, and ecological perspectives that is widely observed in the literature.

### Positioning Within the Innovation Landscape

The PMRT builds on the hackathon model described in a previous study [16], which showed that one-time, intensive events can successfully spark user-centered health innovation by bringing together patients, health care professionals, and developers to co-create digital solutions. The hackathon enabled rapid idea generation, multidisciplinary teamwork, and the development of functional prototypes but also revealed challenges in sustaining project momentum and ensuring long-term implementation after the event. In contrast, the PMRT provides a recurring, structured platform that supports ongoing stakeholder engagement and iterative development. By facilitating continuous collaboration and follow-up, the PMRT addresses the key limitation of the hackathon model, namely, the difficulty of maintaining project progress and impact beyond the initial event.

There have also been multiple initiatives with similar goals as the PMRT. One notable example is the Health Informatics Society of Australia Innovating Health Series in Australia, aimed at advancing digital health innovation by fostering new conversations among health care leaders and industry experts [41,42]. Through a series of roundtables and collaborative activities, Health Informatics Society of Australia sought to challenge existing perspectives and stimulate sector-wide change in response to mounting pressures on the health care system and the rapid evolution of digital technologies. Although the series appears to have concluded in 2020, its emphasis on cross-sector dialogue and co-creation closely aligns with the PMRT’s ongoing, practice-driven approach to stakeholder engagement and innovation in patient monitoring.

The PMRT also shares foundational similarities with international innovation initiatives such as the European Institute of Innovation and Technology (EIT) Health Think Tank Round Table series [43]. EIT Health convenes pan-European policy forums to identify systemic barriers to health care innovation, focusing on regulatory clarity, resource access, and strategic alignment at a macro level. In contrast, the PMRT is a bottom-up, use-case-driven platform embedded in clinical practice, emphasizing clinical relevance, user-centered design, and interoperability. While EIT Health Round Tables are typically yearly, agenda-setting events, the PMRT operates as a recurring ecosystem, producing tangible outcomes such as structured user feedback that inform product development and implementation research. This positions the PMRT as a distinct and complementary format within the landscape of collaborative health innovation.

### Implications for Practice

The PMRT is a well-established initiative that plays a critical role in bridging the gap between clinical practice, research, and innovation development. However, its current format as a local event series inherently limits its broader impact. To enhance its reach and effectiveness, the PMRT concept is being adapted for scalability. This includes transitioning to hybrid event formats and launching an online community platform, thereby facilitating sustained and meaningful exchange beyond the physical roundtable events.

The PMRT model can serve as a foundational element in building communities of clinical innovators, particularly within university hospitals. These innovators, clinicians with intrinsic motivation and interest in digital transformation, are essential for driving organizational change toward a learning health care system [44], as envisioned by the Institute of Medicine. The PMRT provides an effective mechanism for identifying and engaging such individuals and offers a replicable structure that can be adopted by other institutions.

Events like PMRT are more than knowledge exchange platforms; they actively contribute to community building and professional identity formation among digital health pioneers. Organizers and institutions interested in replicating the model are invited to reach out to co-host a local PMRT. The format has proven robust and can be adapted to different clinical environments with minimal effort.

Further research is needed to assess the transferability of the PMRT concept to other regions or thematic areas within digital health. Future developments should consider ways to formalize the evaluation of impact, for instance, by collecting systematic feedback, tracking follow-up collaborations, or studying the implementation of discussed innovations in clinical practice. Additionally, expanding patient and caregiver participation could further strengthen the roundtable’s commitment to inclusive and needs-based digital health development.

### Limitations

There are several limitations to these findings. The first limitation lies in the selected methodological approach and the potential for bias due to the dual roles of several researchers, who were also actively involved in organizing and facilitating the PMRT. Regarding the survey, the sample size was limited to 47 participants, which might restrict the generalizability of the results. In addition, recruitment via LinkedIn likely led to an overrepresentation of industry stakeholders, which may not reflect the full spectrum of perspectives in real-world health care settings. Furthermore, a substantial proportion of participants had attended the PMRT four or more times, indicating that regular attendees might have been more willing to participate in the survey and making it plausible that their responses were biased toward positive experiences. Also, the findings are based on participant-reported perceptions and should therefore be interpreted with appropriate caution, as the inclusion of product-focused sessions and the industry-inclusive nature of the PMRT introduce a potential risk of positive bias. Notably, no responses were received from individuals who may have had negative perceptions of the PMRT, introducing the risk of selection bias. The findings must also be viewed in light of the fact that many participants were already well-versed in patient monitoring, which is beneficial for generating in-depth insights in a focus group setting. However, this limits the findings’ relevance for understanding the needs of newcomers or for informing strategies to introduce and engage new stakeholders in the field of patient monitoring. Finally, the use of a custom-developed, non-validated questionnaire limits psychometric interpretation and comparability with other instruments. We aim to formally validate the questionnaire in future work, including assessment of reliability and construct validity.

### Conclusion

In an era where digital health innovation often struggles to bridge the gap between clinical needs and technological solutions, the PMRT presents a novel, structured low-threshold platform for clinical-centered transdisciplinary dialogue, professional networking, and knowledge exchange. Its strength lies not in broad appeal, but in directly addressing the specific professional realities of its audience, ensuring relevance, trust, and meaningful dialogue. The PMRT’s regular, low-threshold meetings create a stable framework for transdisciplinary exchange, enabling clinicians, researchers, and industry partners to engage on equal footing and identify actionable solutions.

Participant reception confirms high acceptance and perceived impact, with many reporting strengthened community ties, new collaborations, and valuing the interactive workshop format. Its participatory, recurring format fosters sustained engagement and has already led to tangible collaborations between clinicians, researchers, and industry partners. While the PMRT primarily attracts individuals who are already experienced in patient monitoring, this focus has strengthened its role as a trusted environment for high-level discussion and co-creation. To further increase its impact, ongoing efforts aim to broaden participation, especially among nurses and emerging innovators, and expand the initiative through a scalable hybrid model and an online community platform.

## Supplementary material

10.2196/82786 Multimedia Appendix 1 Questionnaire used in the participant survey on the Patient Monitoring Roundtable.

10.2196/82786 Multimedia Appendix 2 List of previous events.

## Abbreviations

BIH: Berlin Institute of Health
ECDF: Einstein Center for Digital Future
EIT: European Institute of Innovation and Technology
GDPR: General Data Protection Regulation
INCH: Initiative for Innovation and Collaboration in Healthcare
PMRT: Patient Monitoring Roundtable
