# Supplementary material for: The Patient Monitoring Roundtable as Catalyst for Health Care Innovation: Case Study
Source: J Particip Med. 2026 Apr 1;18:e82786. doi: 10.2196/82786 (PMC13043008; doi:10.2196/82786)
Supplement: Multimedia Appendix 1 [file jopm-v18-e82786-s001.docx]

**Questionnaire**

1. **I consent to the processing, analysis, and publication of the information provided here in anonymized form, in accordance with applicable data protection regulations, as part of the aforementioned scientific study.**
2. **What is your professional background?**• Physician
   • Nurse
   • Researcher
   • Manufacturer or industry representative
   • Other (free text)
3. **I have experience in patient monitoring and/or other digital health technologies.** (Likert scale)
   • Not applicable at all
   • Rather not applicable
   • Neutral
   • Rather applicable
   • Highly applicable
4. **How many PMRT (Patient Monitoring Roundtable) events have you attended?**• 1
   • 2-3
   • 4 or more
5. **I have already recommended, or would recommend, the PMRT to a colleague.** (Likert scale)
   • Not applicable at all
   • Rather not applicable
   • Neutral
   • Rather applicable
   • Highly applicable
6. **The content of the PMRT events I attended is relevant to my professional interests.** (Likert scale)
   • Not applicable at all
   • Rather not applicable
   • Neutral
   • Rather applicable
   • Highly applicable
7. **The formats used in PMRT events (e.g., small group discussions, workshops) effectively facilitate dialogue among participants.** (Likert scale)
   • Not applicable at all
   • Rather not applicable
   • Neutral
   • Rather applicable
   • Highly applicable
8. **Participating in the PMRT makes me feel part of a community of like-minded professionals in the field of patient monitoring and digital health.** (Likert scale)
   • Not applicable at all
   • Rather not applicable
   • Neutral
   • Rather applicable
   • Highly applicable
9. **As a direct result of attending one or more PMRT events, have you entered into new collaborations?**
   • Yes
   • No
   • Not yet, but I intend to
10. **If you answered "Yes" to question 9, please briefly describe the collaboration(s):**
    • [Free text]
11. **Attending one or more PMRT events has provided me with new insights that I would not have gained otherwise.** (Likert scale)
    • Not applicable at all
    • Rather not applicable
    • Neutral
    • Rather applicable
    • Highly applicable
12. **I will apply new insights or knowledge gained through the PMRT in my professional activities.** (Likert scale)
    • Not applicable at all
    • Rather not applicable
    • Neutral
    • Rather applicable
    • Highly applicable
13. **Which aspects of the PMRT are most relevant to you? (Please rank by relevance)**
    • Informal exchange with clinicians and/or manufacturers
    • Short keynotes
    • Interactive workshops
    • Small group discussions
    • Renowned experts
14. **Were there any barriers you had to overcome to attend PMRT events? If so, which ones?**
    • Scheduling conflicts
    • Technical difficulties (e.g., participation in virtual meetings)
    • Lack of relevant content
    • Family obligations
    • Other
15. **Please indicate your gender:**• Male
    • Female
    • Non-binary
    • Prefer not to say
16. **Please indicate your age group:**
    • 18-29 years
    • 30-39 years
    • 40-49 years
    • 50-59 years
    • 60 years or older
