# Supplementary material for: The Patient Monitoring Roundtable as Catalyst for Health Care Innovation: Case Study
Source: J Particip Med. 2026 Apr 1;18:e82786. doi: 10.2196/82786 (PMC13043008; doi:10.2196/82786)
Supplement: Multimedia Appendix 2 [file jopm-v18-e82786-s002.docx]

**List of previous events**

| Year | Title | Type of Event | Partner Institutions | Estimated number of participants |
| --- | --- | --- | --- | --- |
| 2022 | Patient Monitoring and Alarm Management – Status Quo | Online, Workshop | INCH e.V. | 20 |
| 2022 | Alarm Management – Status Quo | Online, Workshop | INCH e.V. | 20 |
| 2022 | Alarm Management Standards for Patient Monitoring: Reality or Utopia? | Online, Workshop | INCH e.V. | 20 |
| 2022 | Wearables: Clinical Monitoring Device or Lifestyle Gadget? | Online, Workshop | INCH e.V. | 20 |
| 2022 | Charité Pre-Hackathon: Challenges of Patient Monitoring and Alarm Management | Hybrid, Keynote with Workshop | INCH e.V., Stiftung Charité, Berlin Institute of Health, Einstein Center Digital Future | 40 |
| 2022 | Tele-Surveillance: Replacing the general ward? | In-person, Keynote with Workshop | INCH e.V., Berlin Institute of Health, Einstein Center Digital Future | 25 |
| 2022 | User-Testing of Monitoring Devices: Hands-on Workshop | In-person, Keynote and Hands-on Session | INCH e.V., Berlin Institute of Health, Einstein Center Digital Future, Masimo | 25 |
| 2022 | TeleCare Hotel | In-person, Keynote with Workshop | INCH e.V., Berlin Institute of Health, Einstein Center Digital Future | 25 |
| 2023 | Teleccardiology | In-person, Keynote with Discussion | INCH e.V., Berlin Institute of Health, Einstein Center Digital Future | 25 |
| 2023 | Continuous Glucose Monitoring | In-person,  Keynote with Discussion | INCH e.V., Berlin Institute of Health, Einstein Center Digital Future | 25 |
| 2023 | Telemedicine – Panacea or Means to an End? | In-person, Panel Discussion | Event in Cooperation with Healthcare Experience Meetup von IBM iX, INCH e.V. | 40 |
| 2023 | Monitoring in the Hospital of the Future – What Options Are Available Today? | In-person,  Keynote with Discussion | INCH e.V., Berlin Institute of Health, Einstein Center Digital Future | 25 |
| 2023 | Spot-check vs. Continuous Monitoring | In-person, Keynote with Hans-on Session | INCH e.V., Berlin Institute of Health, Einstein Center Digital Future, Masimo | 25 |
| 2023 | User Experience Design in Alarm Management | In-person, Keynote with Workshop, Projekt INALO | Veranstaltung in Kooperation mit denkwerk GmbH, INCH e.V., Berlin Institute of Health, Einstein Center Digital Future | 30 |
| 2023 | Medical Smartwatches – The Future of Intersectoral Patient Monitoring | In-person, Keynote with Hans-on Session | INCH e.V., Berlin Institute of Health, Einstein Center Digital Future, Masimo | 30 |
| 2023 | Two Years of the Patient Monitoring Roundtable Under the Microscope | In-person, Workshop | INCH e.V., Berlin Institute of Health, Einstein Center Digital Future, Masimo | 20 |
| 2024 | Monitoring in Zero Gravity: Insights from Space Research | In-person, Keynote with Discussion | INCH e.V., Berlin Institute of Health, Einstein Center Digital Future, HealthCapital | 30 |
| 2024 | Innovation Together: Participatory Implementation for Digital Healthcare | In-person, Keynote with Workshop | INCH e.V., Berlin Institute of Health, Einstein Center Digital Future, HealthCapital, Masimo, Philips | 30 |
| 2024 | Security in focus: Data protection and cybersecurity in patient monitoring | In-person, Keynote with Workshop | INCH e.V., Berlin Institute of Health, Einstein Center Digital Future, HealthCapital | 30 |
| 2024 | rom Data to Decision: How AI and CDSS Are Transforming Healthcare | In-person, Keynote with Workshop | INCH e.V., Berlin Institute of Health, Einstein Center Digital Future, HealthCapital | 30 |
| 2024 | SDC and the Future of Interoperability in Healthcare | In-person, Keynote with Hans-on Session | INCH e.V., Berlin Institute of Health, Einstein Center Digital Future, HealthCapital, Dräger | 35 |
| 2024 | Understanding Yesterday, Shaping Today: Lessons from the History of Patient Monitoring | In-person, Keynote with Panel Discussion | INCH e.V., Stiftung Charité, Berlin Institute of Health, Einstein Center Digital Future, HealthCapital | 40 |
| 2024 | Patient Monitoring Today: Developments and Current Challenges | In-person, Keynote with Workshop | INCH e.V., Stiftung Charité, Berlin Institute of Health, Einstein Center Digital Future, HealthCapital, Masimo, Philips, Dräger | 40 |
| 2024 | Science or Science Fiction? The Future of Patient Monitoring | In-person, Keynote with Workshop | INCH e.V., Stiftung Charité, Berlin Institute of Health, Einstein Center Digital Future, HealthCapital | 40 |
| 2025 | Patient monitoring and alarm management: adverse events and “collateral damage” | Hybrid, Keynote with Workshops | INCH e.V., Berlin Institute of Health, Einstein Center Digital Future, HealthCapital | 45 in person, 5 online |
| 2025 | Silent innovation: How interoperability is setting new standards in the Silent Patient Room | Hybrid, Keynote with Workshops | INCH e.V., Berlin Institute of Health, Einstein Center Digital Future, HealthCapital, Masimo, Dräger, Philips | 50 in person, 12 online |
| 2025 | Thema: Digital Inclusion for All: Monitoring and Social Implications | Hybrid, Keynote with Workshops | INCH e.V., Berlin Institute of Health, Einstein Center Digital Future, HealthCapital | 25 in person, 4 online |
| 2025 | IT security and data exchange: building blocks for responsible patient monitoring | Hybrid, Keynote with Workshops | INCH e.V., d-fine, Berlin Institute of Health, Einstein Center Digital Future, HealthCapital | 35 in person, 6 online |
| 2025 | Recognize What Matters Early: Experience Situational Awareness in Action | Hybrid, Live Simulation, Keynote with Workshop | INCH e.V., Berlin Institute of Health, Einstein Center Digital , HealthCapital | 35 in person, 8 online |
